# Supplementary material for: Interactome analysis reveals that lncRNA HULC promotes aerobic glycolysis through LDHA and PKM2
Source: Nat Commun. 2020 Jun 22;11:3162. doi: 10.1038/s41467-020-16966-3 (PMC7308313; doi:10.1038/s41467-020-16966-3)
Supplement: Supplementary file 1 — Supplementary Information [file 41467_2020_16966_MOESM1_ESM.pdf]

Supplementary Information

Interactome Analysis Reveals that LncRNA HULC Promotes Aerobic Glycolysis through LDHA and PKM2

C. Wang et al.

Contents

Supplementary Figures 1-7
Supplementary Tables 1-6
Supplementary methods

Supplementary figures

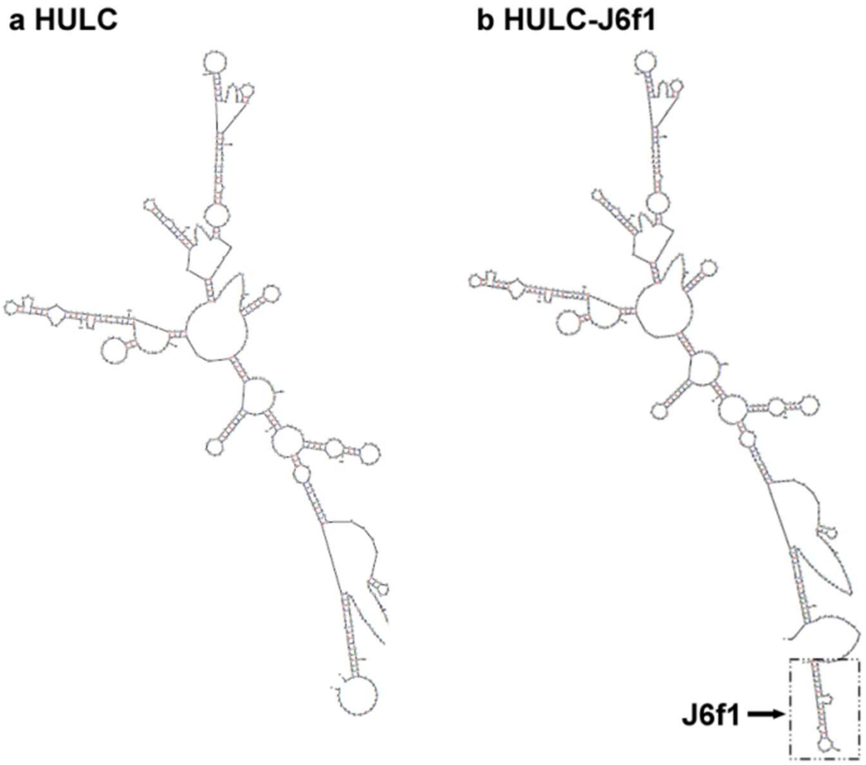

Supplementary Fig. 1 The secondary structures of HULC and HULC-J6f1. The structures were predicted by MFold (http://unafold.rna.albany.edu/).

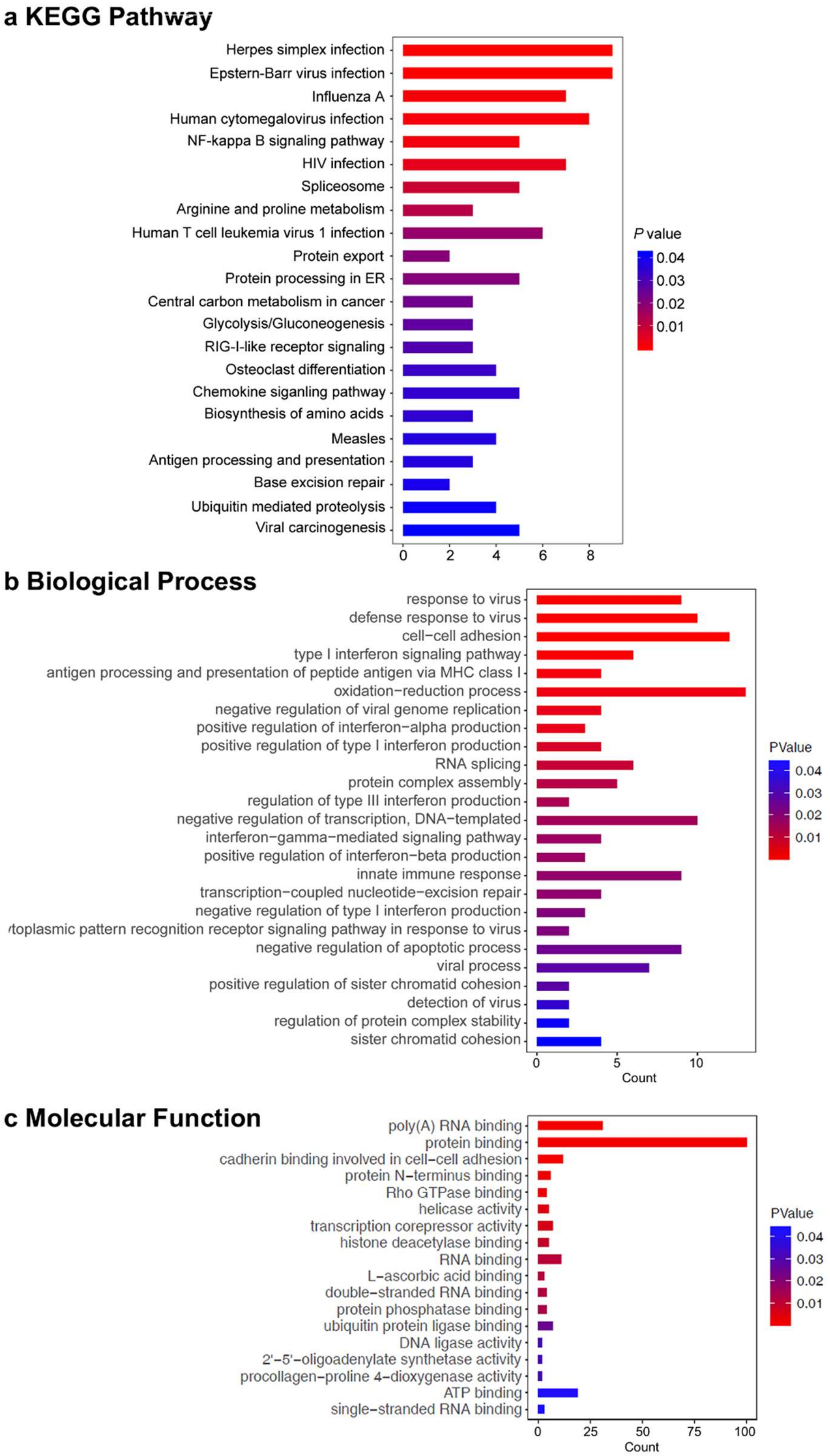

Supplementary Fig. 2 Functional analysis of the HULC interacting proteins. **a.** KEGG pathway analysis of the identified HULC interacting proteins. The analysis was performed by using DAVID (https://david.ncifcrf.gov/). Gene ontology analysis of HULC interacting proteins, including biological process (**b**) and molecular function (**c**).

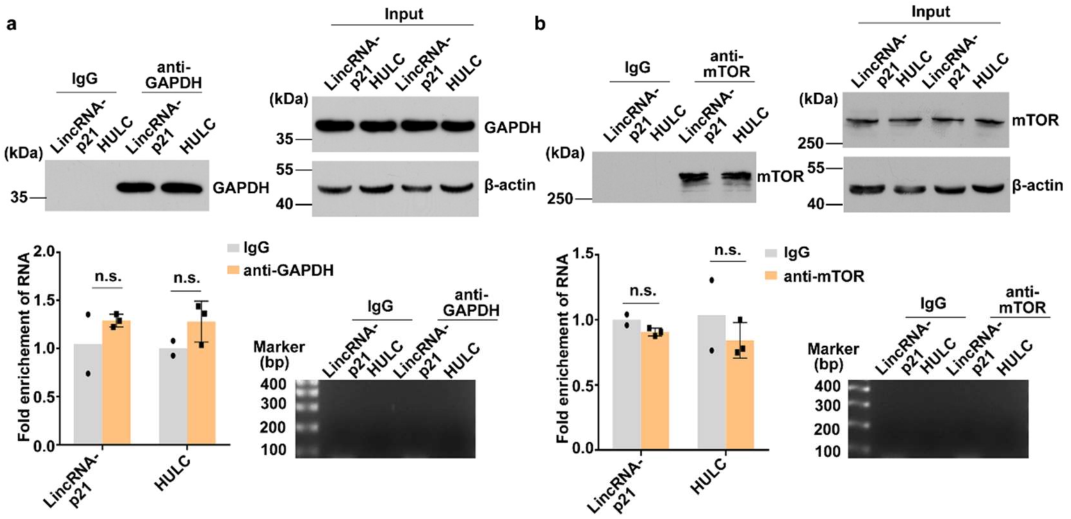

Supplementary Fig. 3 HULC was not enriched in the immunoprecipitates of GAPDH (**a**) and mTOR (**b**). Immunoblots of the target proteins in the cell lysates and immunoprecipitates are shown in the upper panels. The results of qRT-PCR analysis of HULC are shown in the lower panels. LincRNA-p21 was used as a negative lncRNA control. Triplicate independent experiments were performed (note： some values were missing because of the extremely low RNA concentration). Data represent the mean ± s.d. (n.s., not significant, by two-sided Student's *t*-test). Source data are provided as a Source Data file.

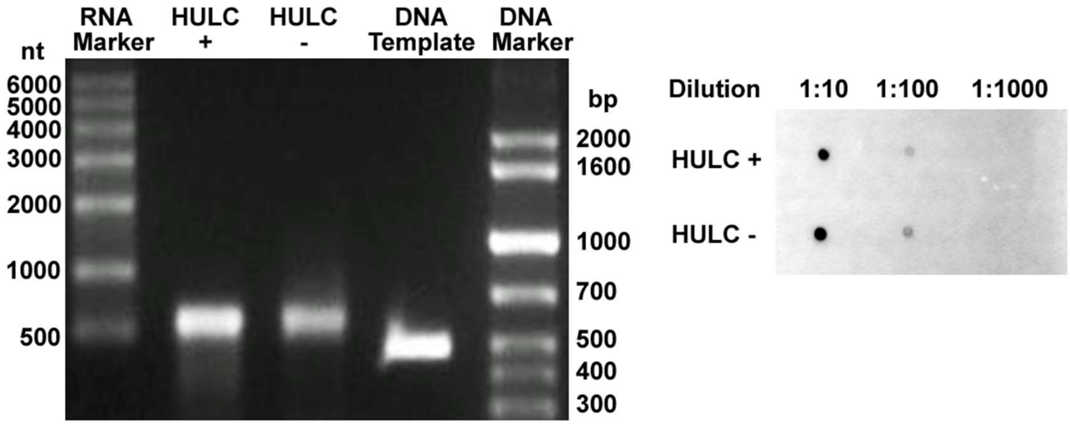

Supplementary Fig. 4 *In vitro* transcription of HULC. Biotinylated HULC and antisense-HULC was synthesized by *in vitro* transcription and examined by agarose gel electrophoresis and streptavidin dot-blot. Source data are provided as a Source Data file.

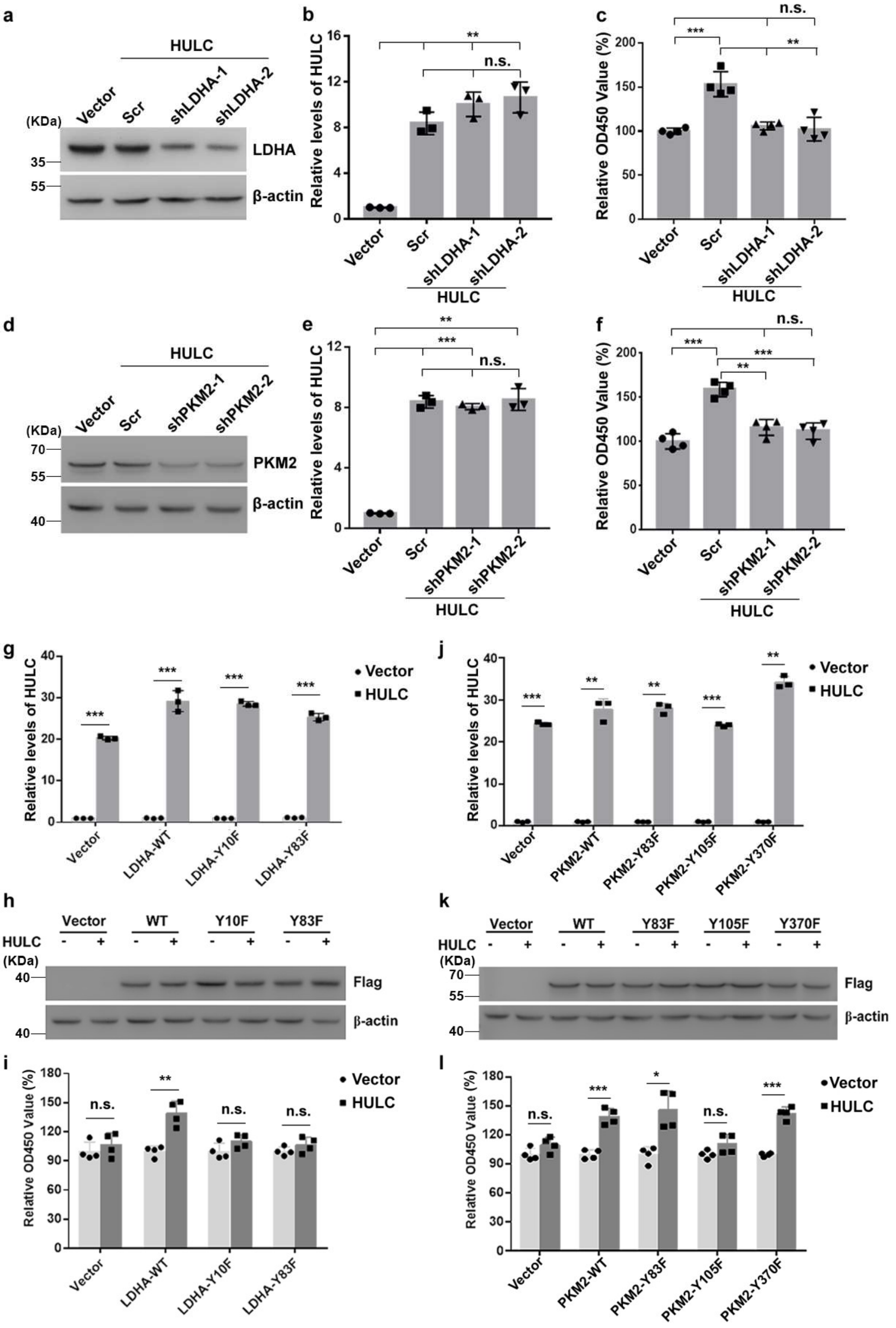

Supplementary Fig. 5 Phosphorylation sites on LDHA and PKM2 responsible for HULC function. **a**, The knockdown of LDHA was validated by Western blotting. **b**, Relative levels of HULC were measured by qRT-PCR. Triplicate independent analyses were performed. **c**, The proliferation of HepG2-HULC cells treated with

shRNAs targeting LDHA was measured by CCK8 assay and compared with the control cells. Four independent experiments were performed. **d**, The knockdown of PKM2 was validated by Western blotting. **e**, Relative levels of HULC were measured by qRT-PCR. Triplicate independent analyses were performed. **f**, The proliferation of HepG2-HULC cells treated with shRNAs targeting PKM2 was measured by CCK8. Four independent experiments were performed. **g**, Relative levels of HULC in HepG2 cells transfected with vector, LDHA-WT, or LDHA phosphorylation mutants, were measured by qRT-PCR. Triplicate independent analyses were performed. **h**, The expression of wild type (WT) LDHA, LDHA-Y10F, and LDHA-Y83F, was measured by Western blotting using anti-Flag antibody. **i**, The proliferation of HepG2 cells expressing LDHA-WT or LDHA phosphorylation mutants was measured by CCK8 assay. Four independent experiments were performed. **j**, Relative levels of HULC in HepG2 cells transfected with vector, PKM2-WT, or PKM2 phosphorylation mutants, were measured by qRT-PCR. Triplicate independent analyses were performed. **k**, The expression of PKM2-WT, PKM2-Y83F, PKM2-Y105F, and PKM2-Y370F, was measured by Western blotting using anti-Flag antibody. **l**, The proliferation of HepG2 cells expressing PKM2-WT or PKM2 phosphorylation mutants was measured by CCK8 assay. Four independent experiments were performed. Statistical analysis was performed by two-sided Student's *t*-test. Data represent mean ± s.d. (*$P < 0.05$, **$P < 0.01$, ***$P < 0.001$, n.s., not significant). Source data are provided as a Source Data file.

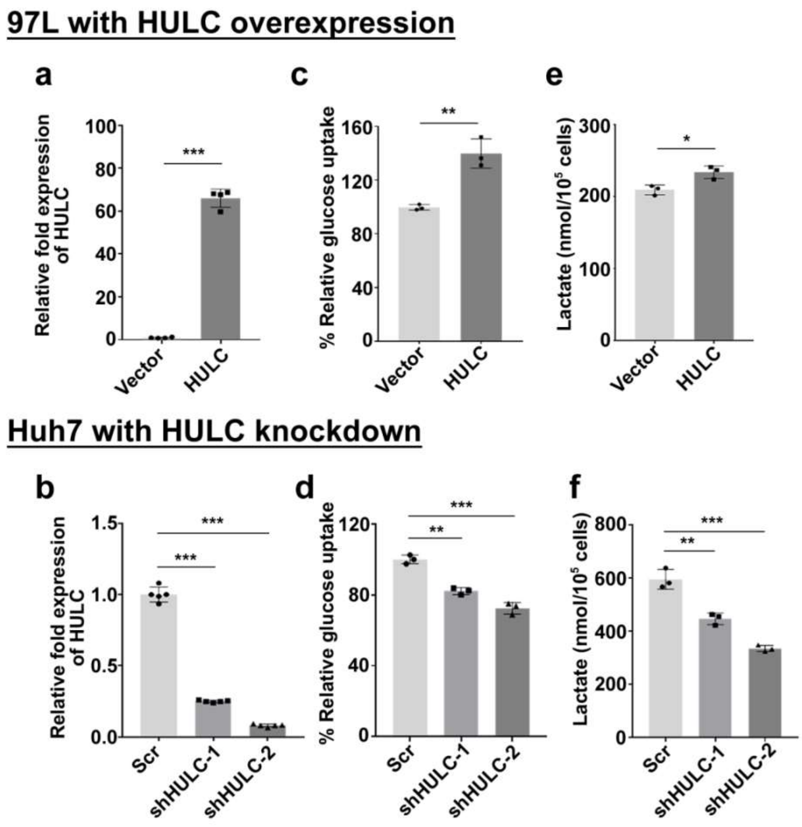

Supplementary Fig. 6 HULC promotes glycolysis in different HCC cell lines. qRT-PCR analysis of HULC in the 97L cells overexpressing HULC (**a**) and Huh7 cells with HULC knockdown (**b**) as compared with corresponding controls. Relative glucose uptake (**c**, **d**) and lactate production (**e**, **f**) were measured and compared with the control cells, respectively. Data represent the mean ± s.d. of triplicate independent analyses (*$P < 0.05$, **$P < 0.01$, ***$P < 0.001$, by two-sided Student's t-test). Source data are provided as a Source Data file.

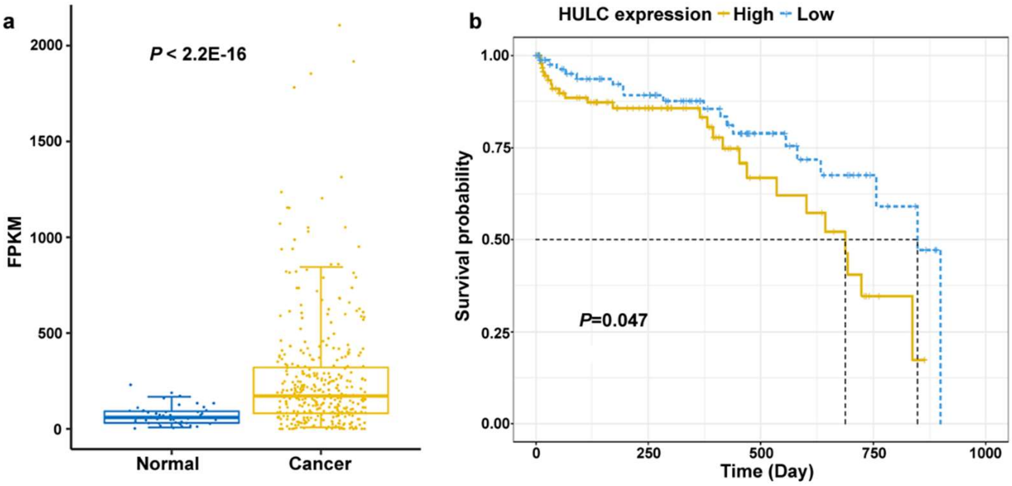

Supplementary Fig. 7 HULC expression in HCC clinical samples. **a**, HULC expression was significantly up-regulated in HCC tumor samples (n= 371) as compared with the adjacent non-tumorous tissues (n = 50). FPKM, fragments per kilobase of exon model per million reads mapped. The data were analyzed by two-sided Student's *t*-test. The box plot includes data between the 25th and 75th percentiles, with the horizontal line representing the median. The upper whisker is the 95th percentile and the lower whisker is the 5th percentile. **b**, Survival rate analysis of the early stage HCC samples with differential HULC expressions (T1, n=129; T2, n=76). The data were downloaded from TCGA database by R package TCGAbiolinks (version 2.10.5), and the analysis was conducted by R package survival (version 2.43.3).

Supplementary Tables

Supplementary Table 1. Anti-sense DNA probes used in RAP-MS experiment.

Probe	5'-3' sequence
Negative control	
LacZ-1	TTTCCCAGTCACGACGTTGTAAAACGACGGCCAGTGAATCCGTAATCATG
LacZ-2	AGTGGCAACATGGAAATCGCTGATTTGTGTAGTCGGTTTATGCAGCAACG
LacZ-3	AGTGCAGGAGCTCGTTATCGCTATGACGGAACAGGTATTCGCTGGTCACT
Anti-HULC	
HULC-1	TCTTCATTCCCTTGTAAAGGCTCCAATTCCATCATGAGTTCCACCCCCAT
HULC-2	CAGATTGGGGCCTTGCTGTATCCTCACGTGGCATAAAGAGAGCTCTTGTC
HULC-3	GATCAGAGTTCCTGCATGGTCTGGTTCTCGTGACGACTCTTCCTGGCTTG
HULC-4	TTAATGTTTTAAACAAAGAATATTCCGGCCTTTACTTCAGAGTTTCTTCC
HULC-5	TAGTTTATTGCCAGGAAACTTCTTGCTTGATGCTTTGGTCTGTTTTGTTT
HULC-6	CCAATTTTTTTCTTTGATAACAACATAATTCAGGGAGAAAGTACAAATTT
HULC-7	TAGTTTTGTTTAATTCTAGTTTCAGTTTTGATGATATTTTGCCATGCTTC

Supplementary Table 2. The list of HULC interacting proteins identified by RAP-MS.

Protein IDs	Gene symbol	Unique peptides	Mol. weight [kDa]	Sequence length	Log_{10}(fold change)	*P* value
P11142	HSP7C	12	70.897	646	1.24	0.033
P00338	LDHA*	5	36.688	332	7.71	0.03
P26599	PTBP1	5	57.221	531	6.947	0.042
P78371	TCPB	5	57.488	535	7.25	0.039
P14625	ENPL	4	92.468	803	0.8	8.39E-05
P30101	PDIA3*	4	56.782	505	7.5	0.001
P38646	GRP75	4	73.68	679	7.521	0.029
O75534	CSDE1	3	88.884	798	6.66	0.018
P07195	LDHB	3	36.638	334	6.72	5.57E-06
P12532	KCRU	3	47.036	417	7.5	0.037
P12956	XRCC6	3	69.842	609	6.76	0.001
Q14204	DYHC1	3	532.4	4646	6.82	0.011
Q99878	H2A*	3	13.936	128	0.73	3.62E-04
P07741	APT	2	19.608	180	6.69	0.002
P12273	PIP	2	16.572	146	0.81	0.017
P21980	TGM2*	2	77.328	687	7.87	0.004
P34897	GLYM	2	55.992	504	6.61	0.017
P62081	RS7	2	22.127	194	6.63	8.65E-07
Q08945	SSRP1	2	81.074	709	6.19	0.049
Q13148	TADBP	2	44.739	414	6.43	0.042
Q15233	NONO	2	54.231	471	7.14	0.027
Q7KZF4	SND1	2	102	910	6.2	0.042
Q8NBS9	TXND5	2	47.628	432	6.49	0.003
Q92597	NDRG1	2	42.835	394	6.59	0.027
Q92734	TFG	2	43.447	400	6.15	0.027

Note: *proteins also observed in the TOBAP-MS experiment. Statistical analysis was performed by two-sided Student's *t*-test.

Supplementary Table 3. Sequences of the DNA primers used for plasmid construction.

Name	Sequence (5'-3')
PKM-exon10	Forward: CCGGAATTCATTGCCCGTGAGGCAGAG
	Reverse: CCGCTCGAGCTGCCAGACTTGGTGAGGAC
PKM-exon9	Forward: CCGGAATTCATAGCTCGTGAGGCTGAGGC
	Reverse: CCGCTCGAGCTGCCAGACTCCGTCAGAAC
LDHA	Forward: CAGTACATCAAGTGTATCATATG
	Reverse: ATTGCAGCTCCTTTTGGATCC
LDHA-Y10F	Forward: GGATCAGCTGATTTTTAATCTTC
	Reverse: GAAGATTAAAAATCAGCTGATCC
LDHA-Y83F	Forward: GGCAAAGACTTTAATGTAACTG
	Reverse: CAGTTACATTAAAGTCTTTGCC
PKM2	Forward: CAGTACATCAAGTGTATCATATG
	Reverse: GGTCTTTGTAGTCCAT GATATC
PKM2-Y83F	Forward: ACTCATGAGTTCCATGCGGA
	Reverse: TCCGCATGGAACTCATGAGT
PKM2-Y105F	Forward: CATCCTCTTCCGGCCCGTT
	Reverse: AACGGGCCGGAAGAGGATG
PKM2-Y370F	Forward: GGACTTTCCTCTGGAG GCT
	Reverse: AGCCTCCAGAGGAAAGTCC

Supplementary Table 4. Interfering sequences used in knockdown experiments.

Name	Sequence (5'-3')
shHULC-1	AATCTGCAAGCCAGGAAGAGT
shHULC-2	AACCTCCAGAACTGTGATCCA
shLDHA-1	GGACTTGGCAGATGAACTTGCCGAAGCAAGT TCATCTGCCAAGTCC
shLDHA-2	GCTGGGAGTTCACCCATTAAGCGAACTTAATG GGTGAACTCCCAGC
shLDHA (UTR)	GAGATCTTGTCCTCTGGAAGCCGAAGCTTCCA GAGGACAAGATCTC
shPKM2-1	GGTGACAGCTTCCTTTCCTGTCG
shPKM2-2	GGGCTGTTGTTCCATTGAAGCC
Scramble sequence	TCCTAAGGTTAAGTCGCCCTC

Supplementary Table 5. Sequences of the DNA primers for qRT-PCR.

Name	Sequence (5’-3’)
HULC	Forward: ACTCTGAAGTAAAGGCCGGAA
	Reverse: TTTGCCACAGGTTGAACACTT
lincRNA-p21	Forward: GGAGACTCTCAGGGTCGAAA
	Reverse: TTAGGGCTTCCTCTTGGAGA
18S rRNA	Forward: GCTTAATTTGACTCAACACGGGA
	Reverse: AGCTATCAATCTGTCAATCCTGTC

Supplementary Table 6. Primer sequences for the sense and antisense *HULC-J6f1* and *HULC* used in the *in vitro* transcription.

Name	Sequence (5'-3')
Sense *HULC-J6f1*	Forward: TAATACGACTCACTATAGGG ATGGGGGTGGAACTCATGATG<br>Reverse: GGCTCAGCACGAGTGTAGCTAAAC
Antisense *HULC-J6f1*	Forward:ATGGGGGTGGAACTCATGAT<br>Reverse: TAATACGACTCACTATAGGGGGCTCAGCACGAGTGTA GCTAAAC
Sense *HULC*	Forward: TAATACGACTCACTATAGGG ATGGGGGTGGAACTCATGATG<br>Reverse: TTTTTTTTTTTTTTTTTTAAGAATGGAC"<br>antisense chain of HULC
Antisense *HULC*	Forward: ATGGGGGTGGAACTCATGAT<br>Reverse: AATACGACTCACTATAGGGTTTTTTTTTTTTTTTTTTAA GAATGGAC"

Supplementary Methods

Identification of endogenous HULC binding proteins by RNA antisense purification and mass spectrometry (RAP-MS)

Probe Design and Synthesis **-** To capture endogenous HULC binding proteins, we designed 5' biotin modified 50-mer DNA oligonucleotides that sequentially covered the sequence of HULC without overlapping. The sequence of each probe was antisense to HULC. To avoid off-target hybridization, we used BLAST to remove sequences that contained a perfect 20 base pair match or an imperfect (90%) 35 base pair match with another transcript or genomic region. Seven probes were selected for HULC, and three probes for *Escherichia coli* lacZ that is absent from human cells was used as negative control (Supplementary Table S1).

UV crosslinking and cell lysate preparation **-** Cells were washed twice with ice-cold PBS. UV-crosslinking was performed on ice with 800mJ/cm^2 at 254nm wavelength in a UV crosslinker. Cells were then scraped off from culture dishes, pelleted by centrifugation at 1,500g for 5min, flash frozen in liquid nitrogen and stored at -80°C. Cells were lysed by ice cold cell lysis buffer (10 mM Tris pH 7.5, 500 mM LiCl, 0.5% dodecyl maltoside, 0.2% sodium dodecyl sulphate, 0.1% sodium deoxycholate) supplemented with protease inhibitor cocktail and RNase inhibitor. The cells were passed 3-5 times through a 27-gauge needle attached to a 1mL syringe in order to disrupt the pellet, and sonicated for 20s in intermittent pulses to shear genomic DNA. Next, samples were treated with 2.5 mM MgCl2, 0.5 mM CaCl2, and 20 U DNase at 37°C for 10 min to digest DNA. Then, the reaction was terminated by 10 mM EDTA and 5 mM TCEP. Equal volume of 2X hybridization buffer (20 mM Tris pH 7.5, 10 mM EDTA, 1 M LiCl, 1% DDM, 0.4% SDS, 0.2% sodium deoxycholate, 8 M urea, 5 mM TCEP) was mixed with the lysate and then incubated on ice for 10min. Lysates were cleared by centrifugation at 16,000g for 10 min and the supernatants were transferred to new tubes.

Isolation of HULC binding proteins by RAP – Lysate from 200 million cells was used for each capture. Biotinylated 50-mer DNA oligonucleotide probes (15 µg per capture) were heat-denatured at 85°C for 3 min and then chilled on ice. Probes were mixed with cell lysate and then incubated at 67°C with intermittent shaking (30 s shaking, 30 s off) for 2h to hybridize probes to the target RNA. Hybrids of biotinylated probes and target RNA were bound to streptavidin beads by incubating each sample with 250 µL of washed streptavidin Dynabeads MyOne C1 magnetic beads at 67°C for 30 min. Beads were washed 6 times with 1X hybridization buffer (10 mM Tris pH 7.5, 5 mM EDTA, 500 mM LiCl, 0.5% DDM, 0.2% SDS, 0.1% sodium deoxycholate, 4 M urea, 2.5 mM TCEP) at 67°C. The washed beads were resuspended in RNase A/T1 elution buffer (20 mM Tris pH 7.5, 2 mM MgCl2) for subsequent processing of the proteins.

Protein identification by mass spectrometry **-** Elution of captured proteins from

streptavidin beads was achieved by digesting the RNA using RNase A/T1 Mix for 2h at 37℃. Beads were magnetically separated from sample using a magnetic stand and the sample supernatant were transferred to a new tube. Each sample was concentrated by using a 10 kDa ultrafiltration device and resuspended in 6M urea (in 50 mM ammonium bicarbonate buffer). The samples were reduced by incubating with 10mM DTT at room temperature for 1h. The reduced proteins were alkylated for 1h in darkness with 40 mM iodoacetamide. The alkylation reaction was quenched by adding DTT to a final concentration of 50 mM. The urea in the solution was exchanged to 50mM ammonium bicarbonate buffer by centrifugation using 10 kDa ultrafiltration devices. Then, trypsin was added at a 50:1 protein to trypsin mass ratio, and the samples were incubated at 37℃ overnight. The digested peptides were analyzed by a Fusion Lumos mass spectrometer as described in the Method section. Three biological replicates were performed, and proteins observed in only one dataset were discarded to ensure the reliability of quantification. Identified proteins with fold change $_{\mathrm{HULC/lacZ}}$ >2 and $P < 0.05$ (by two-sided Student's *t*-test) were considered as potential HULC binding proteins.
