# Supplementary material for: Interactome analysis reveals that lncRNA HULC promotes aerobic glycolysis through LDHA and PKM2
Source: Nat Commun. 2020 Jun 22;11:3162. doi: 10.1038/s41467-020-16966-3 (PMC7308313; doi:10.1038/s41467-020-16966-3)
Supplement: Supplementary file 3 — Reporting Summary [file 41467_2020_16966_MOESM3_ESM.pdf]

nature research

Corresponding author(s): Ruibing Chen, Ning Zhang

Last updated by author(s): May 10, 2020

Reporting Summary

Nature Research wishes to improve the reproducibility of the work that we publish. This form provides structure for consistency and transparency in reporting. For further information on Nature Research policies, see Authors & Referees and the Editorial Policy Checklist.

Statistics

For all statistical analyses, confirm that the following items are present in the figure legend, table legend, main text, or Methods section.

n/a	Confirmed	
☐	☒	The exact sample size (*n*) for each experimental group/condition, given as a discrete number and unit of measurement
☐	☒	A statement on whether measurements were taken from distinct samples or whether the same sample was measured repeatedly
☐	☒	The statistical test(s) used AND whether they are one- or two-sided<br>*Only common tests should be described solely by name; describe more complex techniques in the Methods section.*
☒	☐	A description of all covariates tested
☐	☒	A description of any assumptions or corrections, such as tests of normality and adjustment for multiple comparisons
☐	☒	A full description of the statistical parameters including central tendency (e.g. means) or other basic estimates (e.g. regression coefficient) AND variation (e.g. standard deviation) or associated estimates of uncertainty (e.g. confidence intervals)
☐	☒	For null hypothesis testing, the test statistic (e.g. *F*, *t*, *r*) with confidence intervals, effect sizes, degrees of freedom and *P* value noted<br>*Give P values as exact values whenever suitable.*
☒	☐	For Bayesian analysis, information on the choice of priors and Markov chain Monte Carlo settings
☒	☐	For hierarchical and complex designs, identification of the appropriate level for tests and full reporting of outcomes
☒	☐	Estimates of effect sizes (e.g. Cohen's *d*, Pearson's *r*), indicating how they were calculated

Our web collection on statistics for biologists contains articles on many of the points above.

Software and code

Policy information about availability of computer code

Data collection	Proteomic data in the current study were collected by a LTQ Fusion mass spectrometer (ThermoFisher Scientific, Waltham, MA, USA), and the Xcalibur™ software (version 4.1, ThermoFisher Scientific) was used to set the instrumental parameters and collect the data.
Data analysis	The MaxQuant Software (version 1.6.3.4) was used for database search and quantification analysis.<br>Volcano plot was plotted with the R package ggplot2 (3.1.0).<br>KEGG pathway enrichment analysis was acquired through the R package clusterProfiler (v3.10.1).<br>Known protein-protein interactions (PPIs) were retrieved from String 11 (https://string-db.org/) and integrated in Cytoscape 3.7 for visualization.<br>SPSS version 17.0 software was used for statistical analyses and Graphpad Prism (version 5.0)was used to make plots.<br>ImageJ (version 2.0.0-rc-69/1.52p) was used to measure Western blot band intensities.<br>Seahorse XF24 software (version 2.3.0.19) was used for glycolytic flux analyses.<br>Biacore T200 Evaluation version 2.0 Software and Octet RED96 version 7.0 software were used for binding assays.<br>The RNA sequencing data were downloaded from TCGA database by R package TCGAbiolinks (version 2.10.5), and the analysis was conducted by R package survival (version 2.43.3).

For manuscripts utilizing custom algorithms or software that are central to the research but not yet described in published literature, software must be made available to editors/reviewers. We strongly encourage code deposition in a community repository (e.g. GitHub). See the Nature Research guidelines for submitting code & software for further information.

Data

Policy information about availability of data

All manuscripts must include a data availability statement. This statement should provide the following information, where applicable:

- Accession codes, unique identifiers, or web links for publicly available datasets
- A list of figures that have associated raw data
- A description of any restrictions on data availability

The mass spectrometry proteomic data have been deposited to the ProteomeXchange Consortium via the PRIDE51 partner repository with the dataset identifier PXD012927.
The human protein sequences used for protein identification was downloaded from the UniProt database [https://www.uniprot.org/uniprot/?query=*&fil=organism%3A%22Homo+sapiens+%28Human%29+%5B9606%5D%22+AND+reviewed%3Ayes].
RNA sequencing data were downloaded from The Cancer Genome Atlas (TCGA) database by R package TCGAbiolinks (version,2.10.5) [GDCquery (project = "TCGA-LIHC", data.category = "Transcriptome Profiling", data.type="Gene Expression Quantification", workflow.type = "HTSeq - FPKM")].
The source data underlying Figs 1b-d, 2d, 3a, c-h, 4a, c-i, 5a-e, 6a-f and 7a-j and Supplementary Figs 3a-b, 4, 5a-l and 6a-f are provided as a Source Data file.
All data generated or analyzed during this study are included in this published article (and its supplementary information files).

Field-specific reporting

Please select the one below that is the best fit for your research. If you are not sure, read the appropriate sections before making your selection.

☒ Life sciences ☐ Behavioural & social sciences ☐ Ecological, evolutionary & environmental sciences

For a reference copy of the document with all sections, see nature.com/documents/nr-reporting-summary-flat.pdf

Life sciences study design

All studies must disclose on these points even when the disclosure is negative.

Sample size	No sample size calculation was performed. Sample size was chosen based on established protocols and previous publications in the field (e. g. Nat Commun. 2017, 8:783; Nucleic Acids Res. 2017, 45:9947). At least three independent experiments were performed, the exact numbers of replicates were provided in figure legends.
Data exclusions	No data were excluded from the analyses.
Replication	Experimental findings were successfully reproduced. The number of independent replicates for each experiment was described in the figure legends.
Randomization	For experiments using cell lines, the cells were randomized into experimental groups prior to treatment. For animal study, all the animals were randomly grouped for experiments.
Blinding	The animal experiment was conducted with blinding. Group allocation was performed by one researcher, and the analyses were done by another researcher without knowing the allocation. For cell culture experiments, the investigators were not blinded to group allocation during data collection and analysis, but majority of these experiments were repeated independently by different authors.

Behavioural & social sciences study design

All studies must disclose on these points even when the disclosure is negative.

Study description	*Briefly describe the study type including whether data are quantitative, qualitative, or mixed-methods (e.g. qualitative cross-sectional, quantitative experimental, mixed-methods case study).*
Research sample	*State the research sample (e.g. Harvard university undergraduates, villagers in rural India) and provide relevant demographic information (e.g. age, sex) and indicate whether the sample is representative. Provide a rationale for the study sample chosen. For studies involving existing datasets, please describe the dataset and source.*
Sampling strategy	*Describe the sampling procedure (e.g. random, snowball, stratified, convenience). Describe the statistical methods that were used to predetermine sample size OR if no sample-size calculation was performed, describe how sample sizes were chosen and provide a rationale for why these sample sizes are sufficient. For qualitative data, please indicate whether data saturation was considered, and what criteria were used to decide that no further sampling was needed.*
Data collection	*Provide details about the data collection procedure, including the instruments or devices used to record the data (e.g. pen and paper, computer, eye tracker, video or audio equipment) whether anyone was present besides the participant(s) and the researcher, and whether the researcher was blind to experimental condition and/or the study hypothesis during data collection.*
Timing	*Indicate the start and stop dates of data collection. If there is a gap between collection periods, state the dates for each sample cohort.*

Data exclusions	*If no data were excluded from the analyses, state so OR if data were excluded, provide the exact number of exclusions and the rationale behind them, indicating whether exclusion criteria were pre-established.*
Non-participation	*State how many participants dropped out/declined participation and the reason(s) given OR provide response rate OR state that no participants dropped out/declined participation.*
Randomization	*If participants were not allocated into experimental groups, state so OR describe how participants were allocated to groups, and if allocation was not random, describe how covariates were controlled.*

Ecological, evolutionary & environmental sciences study design

All studies must disclose on these points even when the disclosure is negative.

Study description	*Briefly describe the study. For quantitative data include treatment factors and interactions, design structure (e.g. factorial, nested, hierarchical), nature and number of experimental units and replicates.*
Research sample	*Describe the research sample (e.g. a group of tagged Passer domesticus, all Stenocereus thurberi within Organ Pipe Cactus National Monument), and provide a rationale for the sample choice. When relevant, describe the organism taxa, source, sex, age range and any manipulations. State what population the sample is meant to represent when applicable. For studies involving existing datasets, describe the data and its source.*
Sampling strategy	*Note the sampling procedure. Describe the statistical methods that were used to predetermine sample size OR if no sample-size calculation was performed, describe how sample sizes were chosen and provide a rationale for why these sample sizes are sufficient.*
Data collection	*Describe the data collection procedure, including who recorded the data and how.*
Timing and spatial scale	*Indicate the start and stop dates of data collection, noting the frequency and periodicity of sampling and providing a rationale for these choices. If there is a gap between collection periods, state the dates for each sample cohort. Specify the spatial scale from which the data are taken*
Data exclusions	*If no data were excluded from the analyses, state so OR if data were excluded, describe the exclusions and the rationale behind them, indicating whether exclusion criteria were pre-established.*
Reproducibility	*Describe the measures taken to verify the reproducibility of experimental findings. For each experiment, note whether any attempts to repeat the experiment failed OR state that all attempts to repeat the experiment were successful.*
Randomization	*Describe how samples/organisms/participants were allocated into groups. If allocation was not random, describe how covariates were controlled. If this is not relevant to your study, explain why.*
Blinding	*Describe the extent of blinding used during data acquisition and analysis. If blinding was not possible, describe why OR explain why blinding was not relevant to your study.*

Did the study involve field work? ☐ Yes ☐ No

Field work, collection and transport

Field conditions	*Describe the study conditions for field work, providing relevant parameters (e.g. temperature, rainfall).*
Location	*State the location of the sampling or experiment, providing relevant parameters (e.g. latitude and longitude, elevation, water depth).*
Access and import/export	*Describe the efforts you have made to access habitats and to collect and import/export your samples in a responsible manner and in compliance with local, national and international laws, noting any permits that were obtained (give the name of the issuing authority, the date of issue, and any identifying information).*
Disturbance	*Describe any disturbance caused by the study and how it was minimized.*

Reporting for specific materials, systems and methods

We require information from authors about some types of materials, experimental systems and methods used in many studies. Here, indicate whether each material, system or method listed is relevant to your study. If you are not sure if a list item applies to your research, read the appropriate section before selecting a response.

Materials & experimental systems

n/a	Involved in the study
☐	☒ Antibodies
☐	☒ Eukaryotic cell lines
☒	☐ Palaeontology
☐	☒ Animals and other organisms
☒	☐ Human research participants
☒	☐ Clinical data

Methods

n/a	Involved in the study
☒	☐ ChIP-seq
☒	☐ Flow cytometry
☒	☐ MRI-based neuroimaging

Antibodies

Antibodies used

Mouse monoclonal antibody against β-actin (Cat# T0022; 1:3000) was from Affinity Biosicences (Cincinnati, OH, USA). Rabbit polyclonal antibody against PKM1 (Cat# 15821-1-AP; 1:1000), PKM2 (Cat# 15822-1-AP; 1:30 for IP), LDHA (Cat# 19987-1-AP; 1:30 for IP), LDHB (Cat# 14824-1-AP; 1:2000), TGM2 (Cat# 15100-1-AP; 1:1000), DDX58 (Cat# 20566-1-AP; 1:300), LGALS3BP (Cat# 10281-1-AP; 1:500) and mouse monoclonal antibody against FGFR1 (Cat# 60325-1-Ig; 1:1000) were from Proteintech (Chicago, IL, USA). Rabbit monoclonal antibodies against PKM2 (Cat# 4053S; 1:1000) and GAPDH (Cat# 5174T; 1:1000); rabbit polyclonal antibodies against mTOR (Cat# 2972S; 1:1000), p-LDHA (Y10) (Cat# 8176S; 1:1000) and p-PKM2 (Y105) (Cat# 3827S; 1:1000) were from Cell Signaling Technology (Danvers, MA, USA). Mouse monoclonal antibodies against LDHA (Cat# sc-137243; 1:100 for IF) and PKM2 (Cat# sc-365684; 1:100 for IF) were bought from Santa Cruz Biotechnology (Santa Cruz, CA, USA). Mouse monoclonal antibody against LDHA (Cat# ab85326; 1:50 for IP); rabbit monoclonal antibodies against LDHA (Cat# ab101562; 1:1000), FGFR1 (Cat# ab76464; 1:190 for IP), sodium potassium ATPase (Cat# ab76020; 1:5000), Flag (Cat# 205606; 1:1000) and PDIA3 (also called ERp57, Cat# ab154191; 1:1000); polyclonal antibody against histone H2A (Cat# ab18255; 1:1000); recombinant human PKM2 (rPKM2), LDHA (rLDHA), and FGFR1 intracellular domain (rFGFR1 AA399-822) were bought from Abcam (Cambridge, MA, USA). Alexa Fluor 488 AffiniPure goat-anti-mouse IgG (H+L) (Cat# A-11034; 1:100 for IF) and Alexa Fluor 546 AffiniPure goat-anti-rabbit IgG (H+L) (Cat# A-10040; 1:100 for IF) were from Thermofisher Scientific (Waltham, MA, USA).

Validation

All the antibodies are commercialy available. The application of all the primary antibodies used in this study have been validated by the manufacturers,and used in published articles.
β-actin antibody: Bian WX et al. Nucleic Acids Res 2018. doi: 10.1093/nar/gky1177
PKM1 antibody: Christofk HR, et al. Nature 2008. doi: 10.1038/nature06734
PKM2 antibody: Christofk HR, et al. Nature 2008. doi: 10.1038/nature06734; Wei, et al. Nat Commun 2020. doi: 10.1038/s41467-020-14788-x
LDHA antibody: Li, et al. Cancer Letters 2017. doi: 10.1016/j.canlet.2017.04.034; Li S, et al. Int J Biol Sci 2019.doi: 10.7150/ijbs.30297.
LDHB antibody: Cao, er al. Cancer Research 2019. doi: 10.1158/0008-5472.CAN-18-3842
TGM2 antibody: Wang, et al. Food Funct. 2020. doi: 10.1039/c9fo02491c
DDX58 antibody: Ringeard, et al. Nature 2019. doi: 10.1038/s41586-018-0841-4
LGALS3BP antibody: Silverman, et al. Cancer Research 2012. doi: 10.1158/0008-5472.CAN-11-2165
FGFR1 antibody: Kong, et al. Biochim Biophys Acta Mol Cell Res 2018. doi: 10.1016/j.bbamcr.2018.07.008; Jenks, et al. Cell Rep 2018.doi: 10.1016/j.celrep.2018.05.016
GAPDH antibody: Zhu, et al. Oncol Lett 2020. doi: 10.3892/ol.2020.11445
p-LDHA antibody: Jin, et al. Oncogene 2017. doi: 10.1038/onc.2017.6
p-PKM2 antibody:Angiari, et al.Cell Metab 2020. doi: 10.1016/j.cmet.2019.10.015
Sodium potassium ATPase antibody: Schips, et al. Nat Commun 2019. doi: 10.1038/s41467-018-08026-8
PDIA3 antibody: He, et al. PLoS One 2016. doi: 10.1371/journal.pone.0146530
Flag antibody: Feng, et al. Autophagy 2019. doi: 10.1080/15548627.2019.1570063
Histone H2A antibody: Qin, et al. Nat Commun 2019. doi: 10.1038/s41467-019-09175-0

Eukaryotic cell lines

Policy information about cell lines

Cell line source(s)

HepG2 cells were from American Type Culture Collection (ATCC). Huh7 cells were purchased from the Japanese Collection of Research biosource. MHCC97L cells were gifts from Prof. Zhaoyou Tang.

Authentication

HepG2 cells used in this study were examined for authentication by short tandem repeat (STR) profiling as follows: 1. PCR is amplified with STR Multi-amplification Kit (MicroreaderTM21 ID System); 2. PCR products are assayed with ABI 3730xl DNA Analyzer(Applied Biosystems®). 3. Data were analyzed using GeneMapper3.2 software and then compared with the ATCC and DSMZ databases for reference matching. Huh7 and MHCC97L cells were morphologically examined, and the same batch was used in the previous study (Yun Liu et al. Cancer Res. 2018. doi:10.1158/0008-5472.CAN-17-2432).

Mycoplasma contamination

All cell lines used in this study were tested negative for Mycoplasma contamination.

Commonly misidentified lines
(See ICLAC register)

No commonly mis-identified cell lines were used in this study.

Palaeontology

Specimen provenance	*Provide provenance information for specimens and describe permits that were obtained for the work (including the name of the issuing authority, the date of issue, and any identifying information).*
Specimen deposition	*Indicate where the specimens have been deposited to permit free access by other researchers.*
Dating methods	*If new dates are provided, describe how they were obtained (e.g. collection, storage, sample pretreatment and measurement), where they were obtained (i.e. lab name), the calibration program and the protocol for quality assurance OR state that no new dates are provided.*

☐ Tick this box to confirm that the raw and calibrated dates are available in the paper or in Supplementary Information.

Animals and other organisms

Policy information about studies involving animals; ARRIVE guidelines recommended for reporting animal research

Laboratory animals	Five-week-old female BALB/c-nude mice were used for in vivo tumor growth assay in this study. Housing conditions: 21°C-23°C ambient temperature, 40% humidity, and 12 hours dark/light cycle .
Wild animals	This study did not involve wild animals.
Field-collected samples	This study did not involve samples collected from the field.
Ethics oversight	The experimental protocols were evaluated and approved by the Institutional Review Board of Tianjin Medical University.

Note that full information on the approval of the study protocol must also be provided in the manuscript.

Human research participants

Policy information about studies involving human research participants

Population characteristics	*Describe the covariate-relevant population characteristics of the human research participants (e.g. age, gender, genotypic information, past and current diagnosis and treatment categories). If you filled out the behavioural & social sciences study design questions and have nothing to add here, write "See above."*
Recruitment	*Describe how participants were recruited. Outline any potential self-selection bias or other biases that may be present and how these are likely to impact results.*
Ethics oversight	*Identify the organization(s) that approved the study protocol.*

Note that full information on the approval of the study protocol must also be provided in the manuscript.

Clinical data

Policy information about clinical studies

All manuscripts should comply with the ICMJE guidelines for publication of clinical research and a completed CONSORT checklist must be included with all submissions.

Clinical trial registration	*Provide the trial registration number from ClinicalTrials.gov or an equivalent agency.*
Study protocol	*Note where the full trial protocol can be accessed OR if not available, explain why.*
Data collection	*Describe the settings and locales of data collection, noting the time periods of recruitment and data collection.*
Outcomes	*Describe how you pre-defined primary and secondary outcome measures and how you assessed these measures.*

ChIP-seq

Data deposition

☐ Confirm that both raw and final processed data have been deposited in a public database such as GEO.

☐ Confirm that you have deposited or provided access to graph files (e.g. BED files) for the called peaks.

Data access links<br>*May remain private before publication.*	*For "Initial submission" or "Revised version" documents, provide reviewer access links. For your "Final submission" document, provide a link to the deposited data.*
Files in database submission	*Provide a list of all files available in the database submission.*

Genome browser session
(e.g. UCSC)

Provide a link to an anonymized genome browser session for "Initial submission" and "Revised version" documents only, to enable peer review. Write "no longer applicable" for "Final submission" documents.

Methodology

Replicates

Describe the experimental replicates, specifying number, type and replicate agreement.

Sequencing depth

Describe the sequencing depth for each experiment, providing the total number of reads, uniquely mapped reads, length of reads and whether they were paired- or single-end.

Antibodies

Describe the antibodies used for the ChIP-seq experiments; as applicable, provide supplier name, catalog number, clone name, and lot number.

Peak calling parameters

Specify the command line program and parameters used for read mapping and peak calling, including the ChIP, control and index files used.

Data quality

Describe the methods used to ensure data quality in full detail, including how many peaks are at FDR 5% and above 5-fold enrichment.

Software

Describe the software used to collect and analyze the ChIP-seq data. For custom code that has been deposited into a community repository, provide accession details.

Flow Cytometry

Plots

Confirm that:

- [ ] The axis labels state the marker and fluorochrome used (e.g. CD4-FITC).
- [ ] The axis scales are clearly visible. Include numbers along axes only for bottom left plot of group (a 'group' is an analysis of identical markers).
- [ ] All plots are contour plots with outliers or pseudocolor plots.
- [ ] A numerical value for number of cells or percentage (with statistics) is provided.

Methodology

Sample preparation

Describe the sample preparation, detailing the biological source of the cells and any tissue processing steps used.

Instrument

Identify the instrument used for data collection, specifying make and model number.

Software

Describe the software used to collect and analyze the flow cytometry data. For custom code that has been deposited into a community repository, provide accession details.

Cell population abundance

Describe the abundance of the relevant cell populations within post-sort fractions, providing details on the purity of the samples and how it was determined.

Gating strategy

Describe the gating strategy used for all relevant experiments, specifying the preliminary FSC/SSC gates of the starting cell population, indicating where boundaries between "positive" and "negative" staining cell populations are defined.

- [ ] Tick this box to confirm that a figure exemplifying the gating strategy is provided in the Supplementary Information.

Magnetic resonance imaging

Experimental design

Design type

Indicate task or resting state; event-related or block design.

Design specifications

Specify the number of blocks, trials or experimental units per session and/or subject, and specify the length of each trial or block (if trials are blocked) and interval between trials.

Behavioral performance measures

State number and/or type of variables recorded (e.g. correct button press, response time) and what statistics were used to establish that the subjects were performing the task as expected (e.g. mean, range, and/or standard deviation across subjects).

Acquisition

Imaging type(s)

Specify: functional, structural, diffusion, perfusion.

Field strength

Specify in Tesla

Sequence & imaging parameters

Specify the pulse sequence type (gradient echo, spin echo, etc.), imaging type (EPI, spiral, etc.), field of view, matrix size, slice thickness, orientation and TE/TR/flip angle.

Area of acquisition

State whether a whole brain scan was used OR define the area of acquisition, describing how the region was determined.

Diffusion MRI ☐ Used ☐ Not used

Preprocessing

Preprocessing software

Provide detail on software version and revision number and on specific parameters (model/functions, brain extraction, segmentation, smoothing kernel size, etc.).

Normalization

If data were normalized/standardized, describe the approach(es): specify linear or non-linear and define image types used for transformation OR indicate that data were not normalized and explain rationale for lack of normalization.

Normalization template

Describe the template used for normalization/transformation, specifying subject space or group standardized space (e.g. original Talairach, MNI305, ICBM152) OR indicate that the data were not normalized.

Noise and artifact removal

Describe your procedure(s) for artifact and structured noise removal, specifying motion parameters, tissue signals and physiological signals (heart rate, respiration).

Volume censoring

Define your software and/or method and criteria for volume censoring, and state the extent of such censoring.

Statistical modeling & inference

Model type and settings

Specify type (mass univariate, multivariate, RSA, predictive, etc.) and describe essential details of the model at the first and second levels (e.g. fixed, random or mixed effects; drift or auto-correlation).

Effect(s) tested

Define precise effect in terms of the task or stimulus conditions instead of psychological concepts and indicate whether ANOVA or factorial designs were used.

Specify type of analysis: ☐ Whole brain ☐ ROI-based ☐ Both

Statistic type for inference
(See Eklund et al. 2016)

Specify voxel-wise or cluster-wise and report all relevant parameters for cluster-wise methods.

Correction

Describe the type of correction and how it is obtained for multiple comparisons (e.g. FWE, FDR, permutation or Monte Carlo).

Models & analysis

n/a	Involved in the study
☐	☐ Functional and/or effective connectivity
☐	☐ Graph analysis
☐	☐ Multivariate modeling or predictive analysis

Functional and/or effective connectivity

Report the measures of dependence used and the model details (e.g. Pearson correlation, partial correlation, mutual information).

Graph analysis

Report the dependent variable and connectivity measure, specifying weighted graph or binarized graph, subject- or group-level, and the global and/or node summaries used (e.g. clustering coefficient, efficiency, etc.).

Multivariate modeling and predictive analysis

Specify independent variables, features extraction and dimension reduction, model, training and evaluation metrics.
