# Supplementary material for: Interactome analysis reveals that lncRNA HULC promotes aerobic glycolysis through LDHA and PKM2
Source: Nat Commun. 2020 Jun 22;11:3162. doi: 10.1038/s41467-020-16966-3 (PMC7308313; doi:10.1038/s41467-020-16966-3)
Supplement: Supplementary file 4 — Description of Additional Supplementary Files [file 41467_2020_16966_MOESM4_ESM.pdf]

Description of Additional Supplementary Files

File Name: Supplementary Data 1
Description: The list of all proteins identified in the TOBAP-MS experiment.
